# The war on cancer: have we won the battle but lost the war?

**DOI:** 10.18632/oncotarget.111

**Published:** 2010-06-26

**Authors:** Rachel Brennan, Sara Federico, Michael A. Dyer

**Affiliations:** ^1^Department of Developmental Neurobiology, St. Jude Children's Research Hospital, Memphis, TN 38105; ^2^Department of Hematology/Oncology, St. Jude Children's Research Hospital, Memphis, TN 38105; ^3^Howard Hughes Medical Institute Early Career Scientist

**Keywords:** Translational research, preclinical testing, animal models, cancer, chemotherapy

## INTRODUCTION

In 1971 president Richard Nixon declared a war on cancer and announced his goal to cure cancer by 1976, the bicentennial year. Thirty nine years and more than 100 billion dollars later, the cumulative adult death rate from cancer adjusted for the size and age of the population has improved by less than 5% [1]. In comparison, the death rate for heart disease over this time period has improved by more than 64% [1]. In 2009, the NY Times published a series on the “war on cancer” specifically highlighting some of the suspected causes for these disappointing results. The primary aim of the series was to investigate and discuss the translational research efforts over the past several decades and to explore some of the strategic decisions made by funding agencies as it relates to basic science and clinical research in order to move new therapies into the clinic quickly and safely.

There is no simple explanation for why the death rate due to cancer hasn't improved more than 5% over the past 4 decades. However, the progress made in treating pediatric cancer over the same time period may shed some light on ways to improve our approach to translational research in coming years. Today, the overall cure rate for pediatric cancers approaches 80%; this is a 30% improvement since 1971. This is remarkable when we consider the rarity of pediatric cancer, the limited research funding and lack of investment by the pharmaceutical industry. Most of the progress in improving outcome for pediatric cancer has come from clinical research. Indeed, the majority (>90%) of pediatric cancer patients are enrolled on treatment protocols and there is now abundant evidence that research protocols have helped optimize treatment intensification, drug dosing and timing, chemotherapeutic drug combination, and the identification of prognostic features of disease in relation to treatment plans. In sharp contrast, only 3% of adult cancer patients are enrolled on research protocols [2]. These numbers suggest that the advances in patient outcome for pediatric cancer since the beginning of the war on cancer can be attributed in part to the coordinated participation in clinical research protocols.

## HISTORY OF PEDIATRIC CANCER CLINICAL RESEARCH

The first pediatric cancer cooperative chemotherapy trials were initiated following congressional approval to increase monetary support for the study of cancer-directed chemotherapy in the mid-1950s. Initially these protocols focused on acute leukemia and later expanded to include brain tumors and solid tumors. Patients received chemotherapeutic agents that were shown to have anti-neoplastic properties in vitro or in adult patients. This approach has remained unchanged for the past 5 decades. In pediatric cancer clinical research, we still rely on poorly characterized in vitro and in vivo testing and Phase I, II and III results in adult patients. Initially, drugs were tested individually, but gradually the focus shifted to the evaluation of combination regimens as our understanding of single agent drug resistance mechanisms improved. The current cooperative group of investigators is multidisciplinary in their approach. They use preclinical findings to test new agents, develop novel therapeutic combinations, modify therapeutic schedules, monitor results of ongoing studies, develop patient registries and tissue banks for biological and genomic studies, provide statistical expertise for data analysis and ultimately establish standards of care for disease therapy. This careful and systematic clinical approach has been highly successful, increasing the overall survival for pediatric leukemia from around 10% in the 1950's to 80-90% today.

However, the dramatic improvement in overall survival may overshadow the significant challenges that lie ahead. Patients with high risk and/or rare pediatric cancers have had more limited improvements over the past several decades. Many in pediatric oncology are now coming to the conclusion that we have done as much as we can with existing therapies and drug dose intensification, schedules and other supportive clinical procedures such as autologous bone marrow transplants. Much of the current focus is now on biological studies, genomic studies and targeted therapies. Again, pediatric cancer is uniquely poised to benefit from these emerging approaches to translational research because of the long tradition of clinical and translational research.

Eleven thousand children are diagnosed with cancer annually, which represents less than 1% of all new cancer diagnoses [3]. Among pediatric cancer patients, leukemia accounts for just over 30% of cases with the remainder split between brain tumors and solid tumors [3]. However, brain tumors and solid tumors are diverse, and the complexity of specific diagnoses further complicates clinical research. In addition, the limited number of patients can slow the pace of clinical research trials. The Children's Oncology Group, a worldwide clinical trial cooperative group, runs the largest number of pediatric oncology protocols in the country. Even within this group, it takes a minimum of two years from the time of protocol conception and initial submission to protocol activation and another 3-7 years to complete the trial. When data analysis is factored into the equation, it is not unusual for a clinical research study in pediatric cancer to span an entire decade.

While clinical trials have been essential in advancing the field of pediatric oncology, it is imperative that alternative methods are developed in conjunction with clinical research to improve patient survival. Given the significant time lapse from the formulation of an idea to data accumulation, childhood cancers could benefit immensely from the use of thorough preclinical trials. There is a need to understand the basic molecular, biologic and developmental pathways that lead to formation of these rare tumors. Further, this understanding is essential for the development of targeted anticancer agents that will translate into increased overall survival for pediatric malignancies. Preclinical testing and biology studies have had little impact on childhood cancer patient outcome over the past 5 decades but with the shift toward molecular targeted agents, these efforts have now moved to the forefront of the battle for children's lives in the war on cancer. Retinoblastoma is one example of successful translation of laboratory-based research discoveries into new clinical trials and provides us with a model as we tackle the challenges that lie ahead in the war on cancer.

## 3 PILLARS OF SUCCESSFUL PEDIATRIC CANCER TRANSLATIONAL RESEARCH PROGRAM

The most important consideration for moving new drugs into clinical trials as a result of laboratory-based research is open communication between laboratory investigators and clinical researchers. Without this vital partnership, even the most promising preclinical research studies may never advance to the clinic. Indeed, retinoblastoma represents one such example. Despite decades of research on the RB1 gene and Rb pathway, laboratory based research has had little impact on the clinical management of the disease until recently. While open communication is an essential foundation for a translational research program, it is not sufficient. There are 3 disciplines that are essential for a successful laboratory based research program—preclinical animal models, pharmacology and chemical biology. When these 3 disciplines are integrated into in a diseases-pecific translational research team and partnered with clinical investigators, a tremendous amount of progress can be made in a short amount of time with relatively limited resources.

## PRECLINICAL ANIMAL MODELS

Preclinical animal models that recapitulate the molecular, cellular and genetic features of the human disease are an important starting point for translational research. With recent advances in genetic engineering in mice, it is now possible to delete tumor suppressor genes or ectopically express oncogenes in a variety of cellular lineages during development. This has led to the development of a series of preclinical mouse models for rhabdomyosarcoma, osteosarcoma, neuroblastoma and retinoblastoma among the pediatric solid tumors. Clearly, the genetic lesions that occur in the human tumor should be recapitulated as closely as possible in the mouse model.

A high level of N-myc expression is one of the hallmarks of aggressive neuroblastoma in children. To model this feature of neuroblastoma in children, the tyrosine hydroxylase promoter was used to drive ectopic N-myc expression in the trunk neural crest lineage that populates the adrenal and paraspinal ganglia [4]. These tumors appear to originate in the adrenal or para-adrenal space and mimic many of the features of childhood neuroblastoma. However, even if the genetic lesions are similar to the human disease, this does not necessarily mean that the animal model faithfully recapitulates the human disease. One must also consider the cell biology, molecular signature of the tumors and disease progression. For example, one of the hallmarks of neuroblastoma is catecholamine production and urine catecholamine levels are used as a diagnostic test for patients with neuroblastoma. Using transmission electron microscopy combined with cell biology, it has been shown that the mouse neuroblastomas are indeed catecholinergic based on the presence of dense core vesicles [4]. However, to date, there has not been an unbiased molecular comparison of mouse neuroblastoma to human neuroblastoma and this is important because the mouse tumors do not metastasize at the same frequency or sites as the human disease. Therefore, while neuroblastoma represents a very good example of a mouse model of the human disease, there are still several questions about the mouse tumors in comparison to the human disease that need to be answered. As preclinical testing programs gain prominence and are more widely used for testing new targeted agents, it is important to consider all of the aspects of the human disease not just the initiating genetic lesions.

With a well-characterized mouse model in hand, the next step is to perform the preclinical testing using the same diagnostics and functional assessments that are used clinically for that patient population. It is not appropriate to administer chemotherapy in preclinical models of pediatric cancer and use metrics for efficacy that have no parallel in the clinical setting. Fortunately, most if not all of the tests that are used clinically, are now available for preclinical testing including MRI, PET, CT, ultrasound and a variety of more specialized metrics that are used for specific cancer types. These advances in diagnostic imaging and functional assessment tests in rodents provide unprecedented opportunities to perform comprehensive preclinical testing. Moreover, when combined with the dose and schedule of chemotherapy that is used in patients, it can provide the predictive power that is lacking from the extensively used flank xenografts in immunocompromised mice. We suggest that comprehensive preclinical testing is not only possible for the first time, using sophisticated genetically engineered mouse models of human cancer, but that it is essential to have predictive power for understanding which new combinations of chemotherapy tested in the lab will have the best chance of success in the clinic. In this way, one of the greatest challenges in pediatric cancer (limited patient population), provides us with a tremendous opportunity to take full advantage of laboratory developments in mouse models and diagnostic tools for translational research.

## CHEMICAL BIOLOGY

Several academic research institutes and medical centers now support high-throughput screening infrastructure and chemical biology. Clearly, the goal of such facilities is very different from similar units in pharmaceutical companies but many researchers interested in translational medicine are starting to use high throughput screening for drug discovery and drug development. In many ways, drug discovery and drug development efforts in academia are complementary to efforts in industry. The reason for this is that academic researchers often select targets and research projects for very different reasons than the large pharmaceutical companies. An academic researcher may pick a particular target and/or cancer subtype based on the opportunity to shed light on fundamental biological processes in order to advance our understanding of cancer. In contrast, pharmaceutical companies must weigh very different factors in selecting particular drug targets and disease populations—not the least of which is market share. Moreover, the deeper understanding of the fundamental biological processes being targeted clearly reside in academic labs while the expertise in toxicity, pharmacology, drug formulation and drug development lies within industry. We envision a partnership that combines the best of academic chemical biology and therapeutics with the strength of the pharmaceutical industry.

There are several advantages of such a system. If the high throughput screening and chemical biology efforts at an academic center are run like a mid-size pharmaceutical company in terms of quality control, data collection, data analysis, and standardized assays used to measure solubility, permeability, toxicity and stability then these data can be directly transferred to industry when the time comes for further development as a clinical candidate. In general, both groups are seeking the same shared goal to understand if a particular chemical compound targets the desired protein or pathway and if this has efficacy in cellular and preclinical models. Other important considerations are the options for clinical formulations, off-target effects, and export from cells by drug transporters. If a particular compound moves forward to the point that it is a reasonable candidate then the pharmaceutical industry has all of the expertise and infrastructure to formulate and produce the compound using a GMP facility and they will also perform the appropriate toxicity studies by partnering with a GLP provider. Most of the larger pharmaceutical companies have extensive networks around the globe to bring new agents into Phase I and II trials and academic centers cannot match that infrastructure. Looking forward in an era of molecular targeted therapy for cancer, partnerships between industry and academia that takes advantage of their unique strengths will most likely have the greatest impact on improving the outcome for cancer patients. This is particularly true for pediatric cancer with the exception of clinical trials infrastructure. Because pediatric cancers are rare, pharmaceutical companies have overlooked this patient population and clinical trials would have to be part of more academic clinical research efforts in the context of large national and international consortia.

## PHARMACOLOGY

Virtually all oncology drugs are developed for the adult cancer population and are then re-formatted for pediatric use. This involves more than a simple dose-reduction for the smaller subject. Volume of distribution, gastric absorption, liver and renal clearance, and enzymes utilized for metabolism of drugs vary by age across the pediatric population. Thus, great care must be taken when calculating and applying a human pediatric drug dose from a clinically relevant adult dose. While proper determination of drug doses can be complicated within the same species, it can be an incredible challenge and burden between species. For decades scientists have addressed this problem by using species-specific conversion factors, based purely on weight and body surface area, to determine relevant interspecies dosages [5]. The FDA has a dosage calculator published on line to aid in calculating relevant doses. However, drug metabolism and clearance are species specific. Additionally, the liver, kidneys and hematopoietic system between species may have significant differences in their sensitivity to chemotherapeutic agents. None of these factors are taken into account with the use of the species-specific dose calculations. Therefore, a more appropriate dosing method should be used to determine interspecies doses, specifically one that relies on comprehensive pharmacokinetic and pharmacodynamic studies in which the area under the curve (AUC) is determined for each individual drug over time within a species and then directly applied to the other species.

The advantage to using the species-specific conversion factor is that it is quick and does not require labor and time intensive pharmacokinetic studies. Additionally, this conversion factor can occasionally yield an equivalent drug dose as determined by AUC guided dosing. For example a common pediatric dose of vincristine is 1.5mg/m2. With the aid of the species-specific conversion factor the equivalent adult mouse dose is 0.5mg/kg. This is very similar to the determined dose when using pharmacokinetic studies. In 2002, Groninger and colleagues evaluated the pharmacokinetics of vincristine in children diagnosed with ALL and determined that the median AUC for a dose of 1.5mg/m2 was 0.12uMxh [6]. In 1999 Thompson et al studied the pharmacokinetics of 1mg/kg vincristine in rodents and determined the AUC was 0.32uMxh [7]. Thus, given the linear relationships, the equivalent mouse dose calculated for a human dose of 1.5mg/m2 is equal to 0.4mg/kg. This drug dose is very similar to the dose of 0.5mg/kg achieved by using the species-specific calculator.

However, there are numerous examples in which the species-specific conversion dose varies significantly from the AUC guided dose and/or far exceeds the animal's maximum tolerated dose. One such example is carboplatin dosing. A typical carboplatin dose used in pediatrics is 560mg/m2. With the aid of the species-specific conversion factor the equivalent adult mouse dose is 187mg/kg. However, this dose is higher than the LD10 for a mouse [8] and is simply not tolerated. Using the pharmacokinetic works of Newell and VanHennik published on data obtained from children and mice, the equivalent mouse dose for a pediatric dose of 560mg/m2 is 80mg/kg [9,10]. This dose is less than half of the dose calculated using the species specific calculator.

A detailed understanding of the metabolism and clearance of drugs is essential for proper dosing in children as well as the unique physiology of organs involved in metabolism and clearance. More importantly, potential toxicities associated with the pediatric population must also be considered and tested in juvenile animal studies prior to clinical trials. For example, hedgehog pathway inhibitors were tested in juvenile rodents and found to have profound toxicities that were developmental stage specific. The current clinical trials with hedgehog inhibitors in the pediatric population of medulloblastoma patients are focused on older patients in an effort to minimize these devastating developmental defects (ClinicalTrials.gov Identifier: NCT00939484). While developmental pathways such as Notch and Hedgehog provide attractive targets for pediatric cancer, the broad systemic toxicities may prove to be too detrimental because of the importance of these pathways in normal development and tissue homeostasis.

It is also essential to use pharmacokinetics to identify the appropriate dose and schedule for the preclinical studies. Historically, the vast majority of preclinical studies in rodents for pediatric cancer used doses and schedules of drug administration that were not clinically relevant.

The process of translating preclinical rodent toxicology trials into pediatric clinical protocols has relied upon a number of retrospective reviews in which one-tenth of the mouse LD10 (reported as mg/m2) was found to represent a safe phase I trial starting dose [5,11,12,13,14]. However, these preclinical animal trials all relied on single-dose or single course (i.e. daily x 5 days) administration of a drug. Performing toxicology studies over 2-4 weeks or up to 2 courses in order to check the safety of a proposed starting phase I trial starting dose when given by repeated administration is recommended by the Committee for Proprietary Medicinal Products (CPMP) prior to starting a phase I clinical trial. However, this minimal expansion cannot compare to the lengthy exposure and duration of treatment required in pediatric trials. Further expansion with repeated dosing that mirrors a clinical trial (up to six months) is only required for Phase II, Phase III or Marketing Applications [15]. In addition to the limited duration of study in mouse models, these drugs are most often tested as single agents. While information on the efficacy of a new drug is important, drugs are most often given in combination in the clinical setting. Potential synergy or even antagonistic effects of multi-drug combinations are of vital importance when considering implementation of a new pediatric clinical trial.

In some cases, initiation of phase I trials at one-tenth the mouse MTD/LD10 would have exceeded the human MTD (fludarabine [16],tallimustine [17]). On the other hand, starting with too conservative a dose can result in lengthy dose escalation and delays in further therapeutic trials, as well as the unnecessary use of clinical resources and large number of patients treated with doses that are not therapeutic. A review in 1999 compared 25 cytotoxic chemotherapeutic compounds tested in preclinical murine toxicology studies and later in clinical phase I trials [18]. In 20 drugs where DLT was observed at the human MAD, the ratio of the human MAD to mouse MTD/LD10 was 2.6 (range 0.2-16) and the number of dose escalations required to reach a MAD or DLT was 8 (range 3-19) [18]. In 2010, LeTourneau et al reviewed the literature supporting the choice of starting dose for molecularly targeted agents and found that, while the dose used in phase I trials was overall safe, the section was based on diverse practices and a wide variety of toxicologic parameters without any standardization [19]. In addition, the authors commented that the non-hematologic DTL common in molecularly targeted agents may not be readily predictable with basic pharmacology data alone due to off-target effects. These reviews highlight the variability of inter-species response to chemotherapeutic agents. Even the expansive Pediatric Preclinical Testing Program launched in the early 2000s to identify new agents for therapy in pediatric cancer has focused on testing single agents in short duration dosing schedules [20]. There is a clearly apparent need for the integration of pharmacokinetic and pharmacodynamic investigations with expanded preclinical toxicology studies, both in terms of duration of therapy and use of multi-agent combination therapy.

Currently, most chemotherapeutic regimens utilize complex combinations of drugs. Therefore it is important to also consider how these drugs interact with respect to toxicities and drug absorption and clearance. We have shown previously that the combination of topotecan and carboplatin was more effective than the standard of care in preclinical models of retinoblastoma [21]. However, these two drugs cannot be administered concurrently by the systemic route of administration because of their overlapping toxicity profiles. In this case, local delivery of one agent by subconjunctival injection is the preferred route of drug delivery because the tumor cells will be exposed to both drugs simultaneously but the systemic exposure of the drug delivered by subconjunctival injection is low enough to mitigate systemic toxicities normally associated with the combination chemotherapy. Solid tumor patients would benefit tremendously from improvements in local drug delivery.

## CONCLUSIONS

There have been truly remarkable advances in our understanding of cancer genetics and cancer biology over the past four decades since Nixon declared the war on cancer. In addition, there has been progress in development of new drugs for cancer therapy and complementary efforts in advancing our understanding of cancer etiology, environmental factors, biomarkers and cancer screening. Each of these battles in isolation can be viewed as victories. Further advances in biology, genetics, cancer diagnostics, biomarker development and clinical research will clearly continue to impact patient outcome but without better integration across clinical research and laboratory research questions may remain about where we stand in the war on cancer.

Even with more limited resources, little investment from the pharmaceutical industry and a small patient population, children with cancer have fared much better in the war on cancer. One reason for this has been the focus on a well-coordinated multidisciplinary approach to clinical research. However, clinical research is not enough to continue on this trajectory for pediatric cancer. It would be a mistake to assume that the same strategy will continue to reap the benefits over the next several decades. Most of the oncology drugs in clinical development are targeted therapies. Our ability to match molecular targeted agents to particular cancers and stages of disease and to combine them effectively with broad-spectrum chemotherapy relies on outstanding biology studies and preclinical testing combined with chemical biology and pharmacology—the 3 essential pillars of a successful translational research program. This is true for pediatric cancer and adult cancer. By building on the tradition of coordinated clinical research and focusing our efforts to bridge the gap between clinical research and laboratory based translational research we may be able to make progress towards winning the war on cancer in children and adults.
